# Calpain Small Subunit Mediated Secretion of Galectin-3 Regulates Traction Stress

**DOI:** 10.3390/biomedicines12061247

**Published:** 2024-06-04

**Authors:** Imjoo Jang, Shalini Menon, Indrajyoti Indra, Rabiah Basith, Karen A. Beningo

**Affiliations:** Department of Biological Sciences, Wayne State University, Detroit, MI 48202, USA; yamjoo@gmail.com (I.J.);

**Keywords:** cell migration, traction force (TF), calpain 4 (Capn4), galectin-3 (Gal3), c-Abl kinase, focal adhesion

## Abstract

The complex regulation of traction forces (TF) produced during cellular migration remains poorly understood. We have previously found that calpain 4 (Capn4), the small non-catalytic subunit of the calpain 1 and 2 proteases, regulates the production of TF independent of the proteolytic activity of the larger subunits. Capn4 was later found to facilitate tyrosine phosphorylation and secretion of the lectin-binding protein galectin-3 (Gal3). In this study, recombinant Gal3 (rGal3) was added to the media-enhanced TF generated by *capn4^−/−^* mouse embryonic fibroblasts (MEFs). Extracellular Gal3 also rescued defects in the distribution, morphology, and adhesive strength of focal adhesions present in *capn4^−/−^* MEF cells. Surprisingly, extracellular Gal3 does not influence mechanosensing. c-Abl kinase was found to affect Gal3 secretion and the production of TF through phosphorylation of Y107 on Gal3. Our study also suggests that Gal3-mediated regulation of TF occurs through signaling pathways triggered by β1 integrin but not by focal adhesion kinase (FAK) Y397 autophosphorylation. Our findings provide insights into the signaling mechanism by which Capn4 and secreted Gal3 regulate cell migration through the modulation of TF distinctly independent from a mechanosensing mechanism.

## 1. Introduction

The orchestration of adhesion events during the migration of a cell requires coordinated cues from both intracellular and extracellular factors [1,2,3,4]. The extracellular matrix (ECM) provides cues that are both chemical and physical in nature. Perception of the extracellular physical cues by a cell is referred to as mechanosensing, which is then translated via mechanotransduction into a mechanoresponse [5,6,7]. Evidence suggests that the sensing and force production mechanisms are linked [8,9,10]. For instance, substrate stiffness, in which the cell is exposed to homeostatic tension, can affect the traction forces generated by the actomyosin cytoskeleton of the cell [8,11,12,13,14]. The process by which mechanosensing elicits a change in traction force is poorly understood.

Our group has previously discovered that the proteolytic holoenzymes calpains 1 and 2 are important for mechanosensing, but their individual knockdowns do not show an effect on the generation of traction force. However, we have found evidence that calpain small subunit 1 (Capn4), the common regulatory subunit of the holoenzymes, functions independently of the large subunits in the generation of traction force [15].

To further understand how the calpain 4 (Capn4) subunit regulates the traction force pathway, we previously looked at differential tyrosine phosphorylation levels of cellular proteins from wild-type mouse embryonic fibroblast (MEF) and *Capn4*-silenced MEF cells. We determined that the protein galectin-3 (Gal3) had reduced tyrosine phosphorylation levels in the absence of Capn4 [16]. Gal3 is a lectin-binding protein and is an atypical member of the galectin family of proteins [17,18]. Gal3 is unique amongst family members in that its carbohydrate recognition domain binds to the proline and glycine-rich N-terminal domain of other Gal3 molecules allowing for extracellular oligomerization [19,20]. Three of its tyrosine residues, 79, 107, and 118, were identified as the residues that are phosphorylated, potentially by c-Abl kinase [21,22]. Gal3 has numerous functions both within and outside the cell indicating that its localization is important for each of its roles [18,23,24,25,26,27,28]. Gal3 influences cell adhesion and migration extracellularly in various ways [29,30,31,32,33,34]. Extracellular Gal3 regulates oligomerization and lateral mobility of integrins [32]. Through integrin activation, Gal3 binds to specific galactose-rich N-glycans modified by Mgat 5 (β 1,6 N-acetylglucosaminyltransferase V) at the cell surface that leads to ECM remodeling [34], stabilizes focal adhesion dynamics, and mediates EGF (epidermal growth factor) signaling in cooperation with phosphorylated caveolin-1 [30,33]. Gal3 is secreted by the non-classical pathway, and a recent study has identified the presence of Gal3 in exosomes [35,36,37,38,39]. We have found that in the absence of Capn4, the secretion level of Gal3 was greatly reduced [16]. Physiologically, Gal3 is implicated in fibrotic diseases of the lung, liver, and cardiovascular tissues [40,41] amongst others. The Capn4-mediated regulation of Gal3 and its regulation of traction force used by fibroblasts could be used to remodel the ECM and would provide greater insights into these diseases.

Having identified a connection between Capn4 and the secretion of Gal3, here we asked if Gal3 is linked to the generation of traction force. To this end, we measured traction stress and adhesion strength and compared numbers and morphology of focal adhesions, migration speed, and persistence in the presence and absence of extracellular recombinant Gal3 (rGal3) added to calpain-deficient cells. We also assessed the ability of the cell to sense both static and transient mechanical cues in the presence of rGal3 and its effect on levels of β1 integrin activation and focal adhesion kinase (FAK) auto-phosphorylation. We further identified a kinase that regulates Gal3 secretion and the production of traction force. Furthermore, we identified an essential tyrosine residue in Gal3 that is required for its secretion and subsequent effect on the production of traction force. In summary, we have identified several significant components of a pathway that allows a cell to alternate between the generation of forces and the sensing of the mechanical environment.

## 2. Materials and Methods

### 2.1. Cell Culture and Reagents

All cell lines used in this study including wild-type mouse embryonic fibroblasts (MEFs), calpain 1 and calpain 2 silenced MEFs, and *capn4^−/−^* MEFs obtained from Dr. Peter Greer (Queens University) have been previously described [16,42,43,44]. Cells were cultured in Dulbecco’s Modified Eagle’s Medium (DMEM, high glucose) (Sigma, St. Louis, MO, USA) supplemented with 10% fetal bovine serum (Hyclone, Logan, UT, USA) and 1% penicillin/streptomycin/glutamine (Gibco, Thermo Fisher, Greenville NC, USA) and grown at 37 °C under 5% CO_2_ in a humidified cell culture incubator. Cells were passaged using 0.1% Trypsin–EDTA (Invitrogen, Carlsbad, CA, USA) and were not allowed to exceed eight passages for any given cell line. The secretion of Gal3 was tested by collecting conditioned media from two 60 mm culture dishes at 80% cell confluency. The collected conditioned media were then loaded onto a 4–20% gradient Tris–HEPES–SDS precast polyacrylamide gel system (Pierce Scientific, Rockford, IL, USA) for Western blot or for Coomassie Blue staining to ensure equal loading. Lyophilized recombinant Gal3 (rGal3) was purchased from R&D Systems and reconstituted at 250 μg/mL following the manufacturer’s protocol. For all experiments, rGal3 was added at a 2 μg/mL concentration. For Capn4 and Gal3 gene knockdowns, siGENOME SMARTpool siRNA from Dharmacon RNAi Technology (Thermo Scientific, Greenville, NC, USA) was used. Off-target siRNA, also from Dharmacon RNAi Technology, was used as a control whenever applicable. Appropriate RNA was introduced into cells by nucleofection performed using an Amaxa Nucleofector II device and MEF-compatible nucleofection reagents (MEF 2 nucleofector solution, Lonza, Hayward, CA, USA). pEGFP-N3, pECFP-Gal3, and pEGFP-Gal3 Y107F were kind gifts from Dr. Avraham Raz (Karmanos Cancer Institute, Detroit, MI, USA). Plasmids were stably expressed in MEF cells after nucleofection as described above. MEF cells were treated with 10 μM of Abl kinase inhibitor STI571 (Cayman Chemical, Ann Arbor, MI, USA) or equivalent volume of DMSO. Primary antibodies used for immunofluorescence and Western blot included mouse anti-vinculin monoclonal antibody (V 4505, Sigma, St. Louis, MO, USA), rabbit anti-FAK [pY397] polyclonal antibody (44–624G, Invitrogen, Carlsbad, CA, USA), rat anti-Gal3 monoclonal antibody (gift from Dr. Avraham Raz, Karmanos Cancer Institute), and mouse anti-glyceraldehyde-3-phosphate dehydrogenase (GAPDH), monoclonal antibody (MAB374, Millipore, Burlington, VT, USA) as the loading control. The secondary antibody used for immunofluorescence was Alexa Fluor^®^ 488 goat anti-mouse IgG (H+L) (A11001, Invitrogen, Carlsbad, CA, USA). ECL™ anti-mouse and anti-rabbit IgG, HRP-linked whole antibodies (NA931 and NA934, respectively, GE Healthcare, Piscataway, NJ, USA) were used as secondary antibodies for Western blot experiments. For immunofluorescence, experiments, actin was visualized using Alexa Fluor^®^ 546 phalloidin (A22283, Invitrogen, Carlsbad, CA, USA).

### 2.2. Traction Force Microscopy and Analysis

Cells were seeded on flexible 5% acrylamide and 0.08% N,N-methylene-bis-acrylamide polyacrylamide substrates prepared as described previously [45]. These substrates were coated with fibronectin at a concentration of 5 μg/cm^2^. The estimated Young’s modulus of these polyacrylamide substrates is 1105 Pa. After the cells were allowed to adhere to the substrates by incubating them overnight under regular cell culture conditions, traction force microscopy was performed as described earlier [46]. Briefly, three sets of images were taken per field—a phase contrast image of the cell followed by two fluorescence images of the embedded beads, with and without the cell on the substrate. Bead displacement maps and the cell and nuclear boundaries were then used to calculate and render traction stress values using a custom-made algorithm provided by Dr. Micah Dembo (Boston University). The algorithm has been previously described [47,48].

### 2.3. Mechanosensing Experiments

To study the effect of substrate stiffness on cellular morphology (spread versus round), cells were seeded on polyacrylamide substrates of two varying stiffness achieved by varying the N,N-methylene-bis-acrylamide concentration (0.1% and 0.04% for hard and soft substrates, respectively) keeping the acrylamide concentration constant at 5%. The Young’s modulus of the hard and soft substrates is 1981 Pa and 330.2 Pa, respectively. The substrates were coated with 5 μg/cm^2^ of fibronectin. Cells were seeded and incubated overnight, and then live cell images were taken at 10X magnification. Observed protrusive activity at 40X magnification indicated viability. The number of spread and round cells were observed visually by their area and were then counted from six random fields for each cell line seeded on either of the two substrates. The average cell count was compared.

The effect of a locally applied mechanical stimulus on cell migration was also studied by seeding cells on 5%/0.1% acrylamide/N,N-methylene-bis-acrylamide polyacrylamide substrates coated with fibronectin. The experiment was performed as described previously [45]. Briefly, a blunted microneedle was used to gently push the substrate in front of a migrating cell. This leads to a decrease in the tension within the substrate. Cells respond morphologically to this difference in tension by rounding up or altering their migratory trajectory away from the needle. A “no response” is recorded when it remains on its trajectory toward the needle without any gross morphological change. The response is observed by taking images every 3 min for approximately 1 h.

### 2.4. Cell Adhesion Assay

A centrifugation assay was used to evaluate adhesion strength and has been described previously [15,49]. Briefly, cells were seeded onto 5%/0.08% acrylamide/N,N-methylene-bis-acrylamide substrates coated with fibronectin. The cells were allowed to adhere at 37 °C for 30 min. Adhered cells from 10 random fields were counted before and after centrifugation. Percentage adhesion for each cell line was calculated and compared.

### 2.5. Cell Migration Assay

Cells were seeded onto fibronectin-coated cover glass or 5%/0.08% acrylamide/N,N-methylene-bis-acrylamide polyacrylamide substrates coated with fibronectin and incubated overnight at 37 °C. The migration pattern of a cell was then observed at 40× magnification, and images were collected at 2 min intervals for 2 h. Linear speed (µm/min) and persistence (min) of each cell were then calculated using the custom-built dynamic image analysis system software (DIM version 2.0, Y-L. Wang) based on the x,y coordinates of cell centroids.

### 2.6. Immunofluorescence

Cells were seeded onto fibronectin-coated cover glasses and incubated overnight under regular cell culture conditions. The cells were then fixed and permeabilized using a two-step protocol—first with 2.5% paraformaldehyde (Electron Microscopy Science, Sumpter, SC, USA) at 37 °C for 10 min, followed by a second step with both 2.5% paraformaldehyde and 0.1% Triton X-100 at RT for 10 min. Quenching was performed with 0.5 mg/mL Na BH_4_ (Sigma, St. Louis, MO, USA,) in 1× PBS for 5 min at RT. This was followed by blocking with 5% BSA in PBS for 1 h at room temperature. Following this, an anti-vinculin antibody (Sigma, V4505, St. Louis, MO, USA) was added at a 1:200 dilution and incubated at room temperature for 3 h. Alexa Fluor^®^ 488 anti-mouse secondary antibody (Invitrogen, Carlsbad, CA, USA,) was then added at a 1:500 dilution and incubated for 1 h at room temperature followed by Alexa Fluor^®^ 546 phalloidin (Invitrogen, a22283, Carlsbad, CA, USA,) staining at a 1:500 dilution also for 1 h at room temperature. Each step was followed by PBS washes (3 × 15 min each). Images were then collected using appropriate filters. The number and size of vinculin-containing plaques were then measured using both manual counts and Image J software, version 1.54 (NIH).

### 2.7. Microscopy

Images for all experiments described above were acquired using an Olympus IX81 ZDC inverted microscope fitted with a custom-built stage incubator to maintain cells at 37 °C under 5% CO_2_ for live-cell imaging and a Diagnostic Instruments Boost EM-CCD-BT2000 back-thinned camera. The camera was driven by the IPLab software version 4.0 (BD Biosciences, Franklin Lake, NJ, USA).

### 2.8. Polyacrylamide Gel Electrophoresis and Western Blotting

Each cell line was cultured to 80% confluency in two 60 mm culture dishes coated with fibronectin for protein extraction. Cells were lysed with triple detergent lysis buffer (100 mM Tris-Cl, 300 mM NaCl, 0.5% sodium deoxycholate, 0.2% SDS, 2% NP 40) containing Protease Inhibitor Cocktail (Sigma, St. Louis, MO, USA) and HaltTM Phosphatase Inhibitor Cocktail (Thermo Scientific, Greenville, NC, USA). Protein concentrations were estimated using the Bio-Rad DC protein assay kit. Equal protein concentrations for all cell lines were then loaded onto a 4–20% gradient Tris–HEPES–SDS precast polyacrylamide gel system (Pierce) and resolved at 100 V. The proteins were then transferred using the Bio-Rad semi-dry transfer apparatus onto PVDF membranes (Bio-Rad, Hercules, CA, USA). After transfer, the blots were blocked and then probed with the appropriate antibodies. Rabbit anti-FAK (pY397) polyclonal antibody (Invitrogen, 44–624 G) was used at a 1:1000 dilution in 1% BSA in Tris-buffered saline—0.1% Tween (TBS-0.1% Tween). The antibody incubation was performed overnight at 4 °C. Rat anti-active β1 integrin monoclonal antibody (BD Pharmingen, clone 9EG7), rabbit anti-β1 integrin polyclonal antibody (Millipore, AB1952 Burlington, VT, USA), and goat anti-Arg polyclonal antibody (Santa Cruz, sc-6356) were all used at a 1:500 dilution in 5% non-fat blotting grade milk in TBS-0.1% Tween. Rat anti-Gal3 monoclonal antibody (gift from Dr. Avraham Raz, Karmanos Cancer Institute) was used at 1:500 dilution in 5% non-fat blotting grade milk in phosphate-buffered saline—0.1% Tween (PBS-0.1% Tween). Levels of GAPDH, the loading control, were detected using mouse anti-GAPDH monoclonal antibody (Millipore, MAB374 Burlington, VT, USA) diluted to 1:7000 in 5% non-fat blotting grade dry milk in PBS-0.1% Tween. Commercially available HRP-conjugated secondary antibodies (Amersham) were used and detected with ECL Plus Western Blotting Detection Reagents (Amersham). Washes before and after the secondary antibody treatment in each case were carried out using TBS-0.1% Tween for FAK [pY397], β1 integrin, active β1 integrin, and Arg or PBS-0.1% Tween for Gal3 and GAPDH.

### 2.9. Statistical Analysis

Data were analyzed for the mean and standard deviation and represented by bar graphs in some figures. The two-tailed *t*-test was used to test two variable significances within a hypothesis.

Box and whisker plots were used to represent the mean (the line within the box), the variance (whiskers), and the actual sample points, some of which may be overlapping. Type I errors in the *t*-test *p* values were corrected with the Bonferroni correction formula *p* value = α/σ. Assistance with analysis was provided by Wayne State University, Research Design and Analysis Consulting (RDA).

## 3. Results

### 3.1. Gal3 Is Essential for the Generation of Cellular Traction Force

In a previous study, we discovered that cells deficient in Capn4 are impaired in their ability to produce traction force [15]. Furthermore, this function is unique to the small subunit, as knockdown or suppression of the catalytic activity of the large subunits, calpain 1 and calpain 2, did not affect traction forces. In subsequent experiments, we discovered that Capn4 indirectly alters the tyrosine phosphorylation status of multiple proteins, including the lectin-binding protein Gal3, and does so independently of the catalytic subunits [16]. Although the intracellular expression level of Gal3 remains unaffected in the absence of Capn4, its level of tyrosine phosphorylation is reduced. Furthermore, a deficit in phosphorylation prevents the secretion of Gal3 from the cell. Extracellular Gal3 is known to regulate cell adhesion and migration, although the specifics of its function related to Capn4 are lacking [29,33].

**Figure 1:**
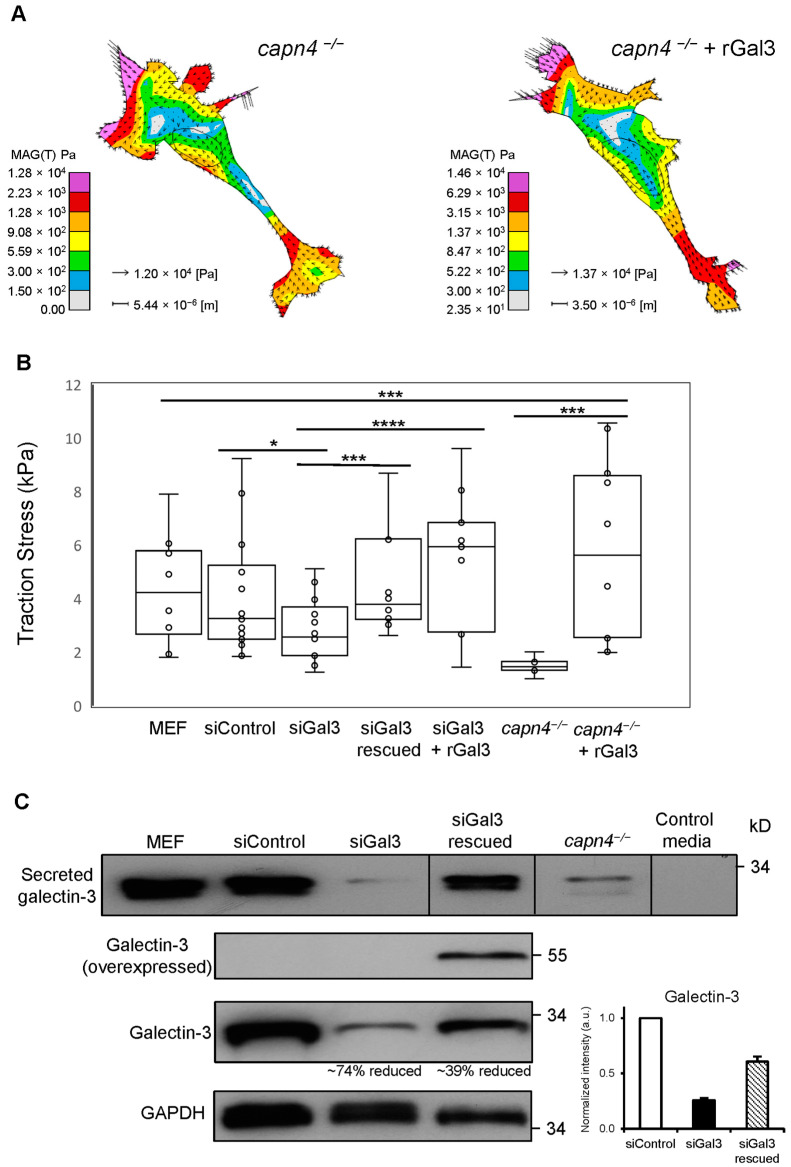
Extracellular Gal3 rescues the defect in traction stress in Gal3 silenced or *capn4^−/−^* MEF cells. (**A**) Representative vector plots depict the magnitude and direction of traction stress exerted by a *capn4^−/−^* MEF cell (*capn4^−/−^*; left) and a *capn4^−/−^* MEF cell with recombinant Gal3 added exogenously (*capn4^−/−^* + rGal3; right). The vectors indicate the direction and magnitude of traction stress. The color map illustrates magnitude. (**B**) The box and whisker graphs of average traction stress exerted by wild-type MEF cells, MEF cells treated with either control siRNA or Gal3 siRNA (siGal3), siGal3 rescued by expressing Gal3 plasmid (siGal3 rescued), siGal3 with recombinant Gal3 added exogenously (siGal3 + rGal3), *capn4^−/−^* and *capn4^−/−^* + rGal3. Data from 8–10 cells were collected for each cell type, typically 2 cells per chamber dish for 5 independent trials were collected. (**C**) Western blot of Gal3 from MEF-conditioned media, siControl, siGal3, siGal3 rescued, *capn4^−/−^*, and control media (top), overexpressed Gal3 and endogenous Gal3 from cell lysates of siControl, siGal3 and siGal3 rescued (two middle), and GAPDH served as the loading control (bottom). A graph indicates endogenous Gal3 levels upon siRNA treatment. Data represent one of three independent trials. Whiskers represent variation in data about the mean. Student’s *t*-test * *p* < 0.05; *** *p* < 0.005; **** *p* < 0.001.

To solidify the functional connection between Capn4 and Gal3, we have asked if extracellular Gal3 influences the magnitude of traction forces produced by migrating fibroblasts. We used traction force microscopy (TFM), performed on polyacrylamide gels of moderate stiffness (Young’s Modulus (E) = 1105 Pa), to measure the magnitude of stress in MEF (Figure S1), *capn4^−/−^* and *capn4^−/−^* + recombinant Gal3 (rGal3) (Figure 1A). Measurements from MEF cells, under conditions of Capn4 and Gal3 deficiencies, were compared to wild-type and non-target siRNA control cells. As in previous experiments, the *capn4^−/−^* MEF cells produced three-fold less traction than the wild-type MEF cells (Figure 1B). Likewise, the silencing of Gal3 resulted in MEF cells with impaired traction, thus mirroring the *capn4^−/−^* traction phenotype. The reduced traction in Gal3-silenced MEF cells was restored by expressing Gal3 plasmid. We observed that the secretion of Gal3 was reduced by 74% in Gal3- and greater than 85% in Capn4-deficient cells (Figure 1C). To address whether the secretion of Gal3 contributed to the shared traction phenotype, we added exogenous recombinant Gal3 (rGal3) to the medium of Gal3-silenced and *capn4^−/−^* cells. The addition of Gal3 not only rescued the defect in traction observed in the Gal3-deficient and *capn4^−/−^* MEF cells, but it enhanced the magnitude beyond that of the control cells (Figure 1B). These results suggest that the defects in traction force observed in Gal3- and Capn4-deficient cells result from a lack of secreted Gal3 and are indirectly mediated by Capn4 itself.

### 3.2. Extracellular Gal3 Affects the Numbers, Localization, Morphology, and Strength of Focal Adhesions

**Figure 2:**
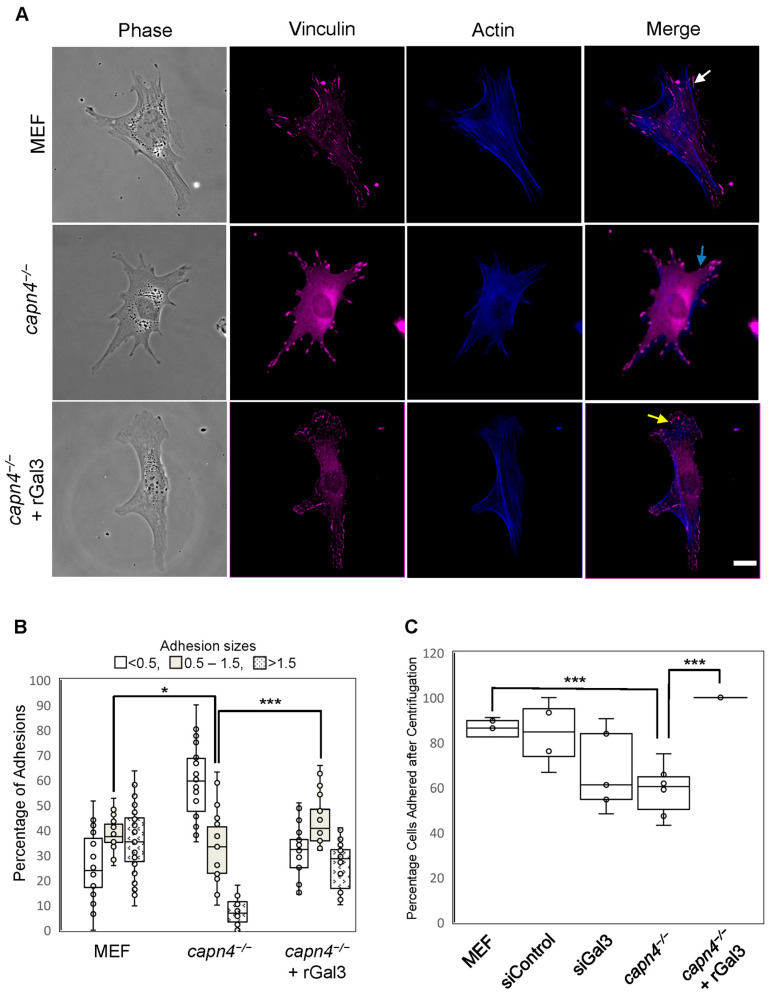
Gal3 mediates changes in size and localization of focal adhesions and enhances adhesion strength. (**A**) Anti-vinculin antibody (magenta) illustrates adhesion locations in wild type MEF cells (top row), *capn4^−/−^* MEF cells (middle row), and *capn4^−/−^* MEF cells treated with rGal3 (bottom row). Similarly, stress fibers are visualized by the staining of actin using fluorescent phalloidin (blue) in MEF, *capn4^−/−^* and *capn4^−/−^* + rGal3 cells (scale bar, 10 µm). The white, blue, or yellow arrows in Merge images indicate representative adhesion in different sizes: >1.5 µm^2^, <0.5 µm^2^, or 0.5–1.5 µm^2^, respectively. (**B**) Box and whisker graphs represent the average number of adhesions within three size ranges (<0.5 µm^2^, 0.5–1.5 µm^2^, or >1.5 µm^2^) in each of the three cell lines. The number of focal adhesions was obtained from 25 to 30 cells for each cell line in three independent staining of duplicate chamber dishes per line, a total of >4000 adhesions. (**C**) Adhesion strength is expressed as a percentage of the number of cells that remain adhered after centrifugation. Data represent three independent trials, each performed in replicate, for each cell line. Whiskers represent the variation in data about the mean. Student’s *t*-test * *p* < 0.05; *** *p* < 0.005.

Capn4-deficient MEF cells exhibit defective focal adhesion maturation [15]. Wild-type MEF cells typically display adhesions of varying sizes ranging from small complexes that form at the edge of the cell and more mature adhesions increasing in size as they grow and move toward the center of the cell [40,41]. However, in *capn4^−/−^* cells, this process is perturbed as indicated by adhesions of uniform sizes that are found at the periphery of the cell, with few found within the cell body. This abnormality in focal adhesion size and localization was also accompanied by a decrease in adhesion strength of the *capn4^−/−^* MEF cells [15]. These defects in adhesions and strength likely contribute to the reduction in traction stress that we see in the absence of Capn4. Since we were able to rescue the traction force defect of *capn4^−/−^* MEF cells by the external addition of rGal3 to the media, we evaluated the effect of extracellular Gal3 on adhesion size, numbers, and strength. To identify size defects, the focal adhesion proteins vinculin and actin were visualized in wild-type, *capn4^−/−^* MEF cells and *capn4^−/−^* cells with rGal3 added to the media. As expected, more mature adhesions (as determined by their size and location) were found in the cell body of wild-type MEF cells when compared to *capn4^−/−^* cells, where the adhesions primarily localized to the periphery of the cell and had poor overlap with actin (Figure 2A). In contrast, when rGal3 was added to the media of the *capn4^−/−^* cells, the number of larger adhesions within the cell body increased greatly and overlap with actin staining was observed (Figure 2A). Indeed, quantification of the size and number of focal adhesions showed a significant increase (*p* < 0.01) in the number of adhesions of 1.5 µm^2^ in *capn4^−/−^* cells treated with rGal3 compared to knockout cells alone (Figure 2B). The numbers of adhesions in *capn4^−/−^* cells with rGal3 added externally had no significant difference from those in wild-type MEF cells. As shown in Figure 2B, *capn4^−/−^* MEF cells had more than 60% of focal adhesions that were smaller than 0.5 µm^2^ (focal complexes) and were localized to the periphery of the cell. These results suggest two things: First, extracellular Gal3 results in focal adhesion sizes and localization similar to wild-type MEF, and second, focal adhesions grow in size in Capn4-deficient cells but a few increase to greater than 1.5 µm^2^ into the cell body. Further studies are required to identify the Gal3-mediated mechanism of focal adhesion growth and turnover.

To test for the strength of adhesiveness to the substrates, we used a previously described centrifugation assay [15,49]. Using the same set of cells described above in addition to Gal3-specific siRNA, we measured cellular adhesion strength. We found that in the absence of Capn4, approximately 60% of the cells remained adhered to the polyacrylamide substrate on which they were seeded (Figure 2C). In comparison, 80–85% of wild-type MEF cells and MEF cells treated with control siRNA remained adhered after centrifugation (Figure 2C). The addition of rGal3 to the media enhanced the adhesiveness of *capn4^−/−^* cells, and more than 95% of cells remained adhered to the substrate (Figure 2C). Silencing of *Gal3* in MEF cells also reduced the adhesion strength to approximately 67%, although the difference is not statistically significant (Figure 2C). Our results indicate that Gal3 in the extracellular environment contributes to strengthening the adherence of focal adhesions.

### 3.3. Extracellular Gal3 Impacts Linear Speed and Persistence of Migration

**Figure 3:**
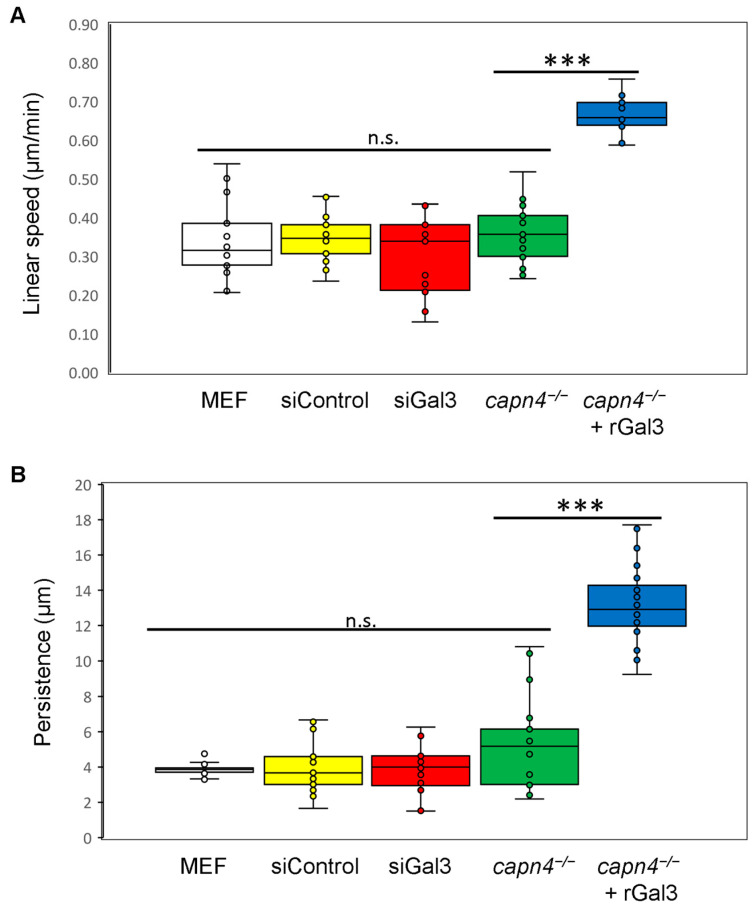
Extracellular Gal3 influences migration speed and persistence. (**A**) Linear speed of wild-type MEF cells (MEF), cells treated with control siRNA (siControl) or Gal3 siRNA (siGal3), *capn4^−/−^* MEF cells (*capn4^−/−^*) and *capn4^−/−^* MEF cells with rGal3 exogenously added (*capn4^−/−^* + rGal3) on fibronectin-coated glass coverslips. (**B**) Persistence of MEF, siControl, siGal3, *capn4^−/−^*, and *capn4^−/−^* + rGal3. An average of 20 cells were observed for each cell type. Each cell was observed for 2 h, and each group of cell types represents an independent trial (4 trials). Whiskers represent the variation in data. Student’s *t*-test; *** *p* < 0.005; n.s., not significant.

Cells are known to migrate individually and collectively [50,51]. Biochemical and biophysical signals, both extracellular and intracellular, can alter the directionality (persistence) and speed of migration [52]. Parameters such as adhesiveness and strength of traction stress can modulate speed and persistence [53]. We measured persistence and the linear speed of wild-type MEF cells and *capn4^−/−^* MEF cells when the cells were seeded on 5 μg/cm^2^ fibronectin-coated glass coverslips (to minimize biophysical communication) or polyacrylamide substrates. We found that on both coated glass (Figure 3A,B) and gels (Figure S2), both linear speed and persistence of *capn4^−/−^* MEF cells were comparable to wild-type MEF cells. We also added rGal3 to *capn4^−/−^* MEF cells cultured on fibronectin-coated coverslips. Surprisingly, both linear speed and persistence were significantly increased upon rGal3 addition, exceeding wild-type and *capn4^−/−^* levels (*p* < 0.005) (Figure 3A,B). However, siRNA-mediated silencing of Gal3 did not affect these parameters, suggesting that even small amounts of secreted Gal3 may suffice to support normal migration speeds and persistence; indeed, previous studies have found low levels of Gal3 may act as an antagonist occupying ECM-binding sites, allowing the cell to alter between migration and remodeling under normal physiological states [50].

### 3.4. Extracellular Gal3 Does Not Rescue the Mechanosensing Defect of Capn4-Deficient Cells

Cells can sense substrate stiffness, topography, and localized mechanical forces generated by neighboring cells through a phenomenon referred to as mechanosensing. There is evidence that different forms of external stimulation produce different responses [6,54,55,56]. Many cell types prefer to spread and migrate on substrates whose stiffness closely matches their endogenous substrate [57,58,59,60,61,62,63]. For example, wild-type MEF cells spread and migrate better on stiffer substrates compared to soft polyacrylamide substrates coated equally with fibronectin [60]. When we tested the calpain-deficient cells on hard and soft substrates (static mechanical input), we found that MEF cells deficient in calpains 1 or 2 could sense a difference in stiffness. They responded similarly to wild-type cells on these same substrates, whereas Capn4-deficient cells could not sense the difference. In contrast, calpain 1, 2, or 4-deficient cells were unable to sense locally applied stimuli provided by pushing on the substrate with a blunted needle, immediately in front of a migrating cell [15]. A wild-type cell responds to the local stimulus by rounding up or changing the migratory trajectory to avoid the stimulus. Given that secreted Gal3 is essential for rescuing defects in traction force and adhesions in the *capn4^−/−^* cells, a process linked to the sensing of external mechanical stimuli, we asked if exogenous Gal3 could rescue the sensing defects of *capn4^−/−^* MEF cells.

**Figure 4:**
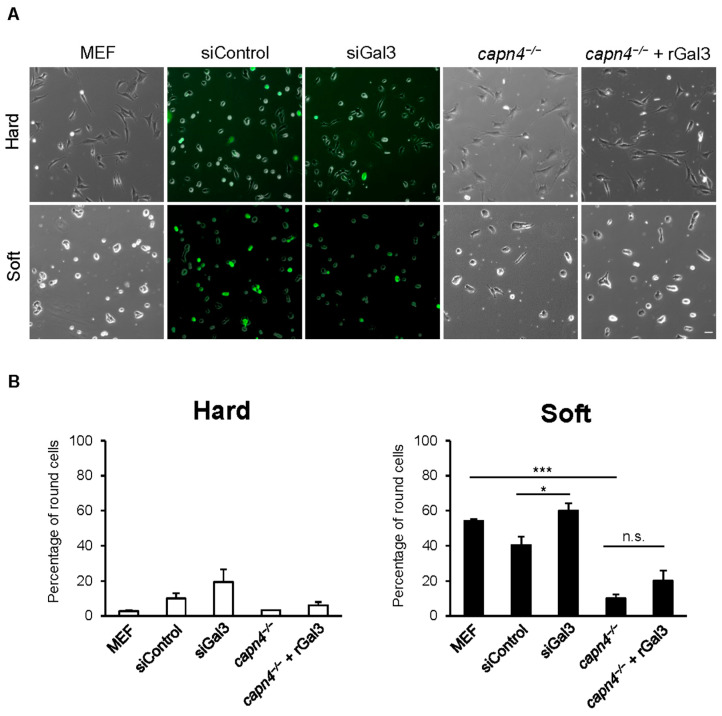
Extracellular Gal3 is not required for sensing the homeostatic tension of the underlying substrate. (**A**) Representative images (10×) of MEF, siControl, siGal3, *capn4^−/−^*, and *capn4^−/−^* + rGal3 show cell morphology of the cells on fibronectin-coated hard and soft polyacrylamide substrates. siRNA-treated cells have been co-nucleofected with a GFP plasmid to ensure that only nucleofected cells are considered during counting (scale bar, 50 µm). (**B**) Bar graphs represent the percentage of round cells when seeded on hard or soft polyacrylamide substrates coated with fibronectin (at least 80 cells were examined for each condition, replicates of 3 trials). Error bars represent mean ± SEM. Student’s *t*-test * *p* < 0.05; *** *p* < 0.005; n.s., not significant.

Using fibronectin-coated hard and soft polyacrylamide substrates, we evaluated the ability of cells to spread normally. As expected, all cell lines seeded on hard substrates responded similarly and 90–95% of the cells were spread as for wild type. However, when plated on soft substrates, almost half the number of wild-type MEF cells and MEF cells treated with control non-target siRNA or Gal3 siRNA remained rounded (Figure 4A,B). It should be noted that siGal3 and its control did register as *p* < 0.043, suggesting a weakly significant difference. This is likely due to the incomplete knockdown, which was observed to be 74% in Figure 1B. However, regarding the sensing experiment, 85% of *capn4^−/−^* MEF cells failed to sense the soft substrate and spread normally. The addition of exogenous rGal3 to *capn4^−/−^* MEF cells failed to rescue this defect and 80% of them spread normally (Figure 4A,B). These results suggest that extracellular Gal3 is not involved in the mechanosensing pathway adopted by cells to sense the static stiffness of its environment.

**Figure 5:**
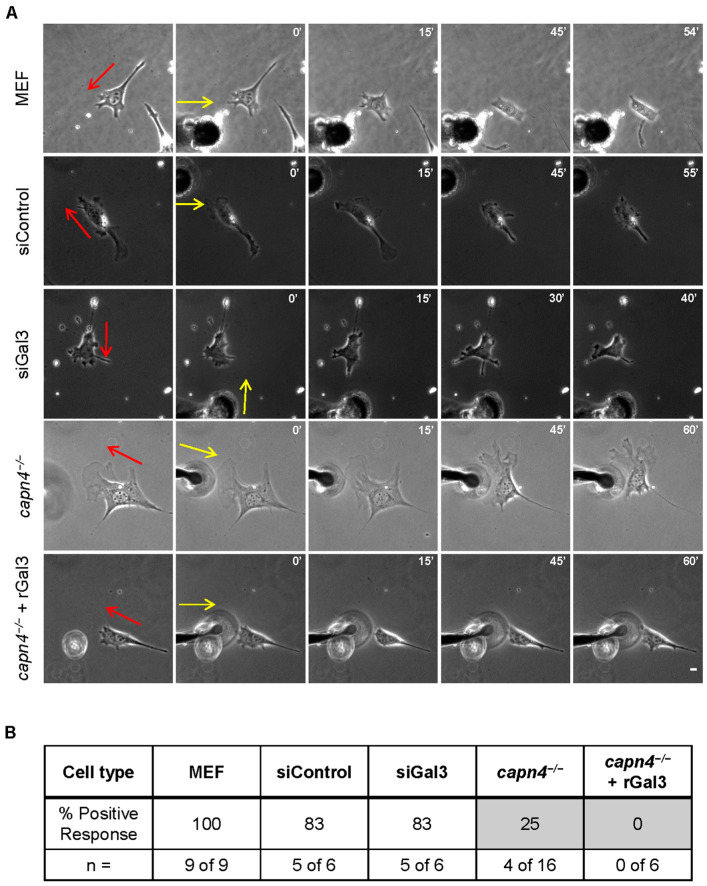
Gal3 is not involved in sensing a locally applied mechanical stimulus. (**A**) Representative time-lapse images display cellular responses of MEF, siControl, siGal3, *capn4^−/−^*, and *capn4^−/−^* + rGal3 in response to an externally applied local mechanical stimulus. The migration trajectories prior to stimulation are indicated by red arrows. The yellow arrows in the second column denote the orientation in which the blunted needle is pushed onto the substrate (scale bar, 10 µm). (**B**) The table summarizes the rate of positive responses (rounding up of the cell or migrating away from the stimulus) observed for each cell type. The number of cells that showed a positive response is indicated, and collection of data from 1 of each cell type was attempted in each of the 10 trials.

To address the ability of *capn4^−/−^* MEF cells treated with rGal3 to sense a locally applied transient stimulus, we subjected these cells to a technically difficult needle-pushing assay described earlier. None of the Capn4-deficient cells treated with rGal3 responded to the stimulus *(n* = 0 of 6) (Figure 5A,B). Similarly, only 4 out of 16 *capn4^−/−^* cells responded to the externally applied local stimulus (Figure 5A,B). However, most of the wild-type MEF cells (n = 9 of 9), control siRNA- (n = 5 of 6), or Gal3 siRNA-treated MEF (n = 5 of 6) cells responded to the applied stimulus (Figure 5A,B). These results suggest that Gal3 is not important for cellular mechanotransduction in response to static tension or a locally applied transient stimulus, although Gal3 clearly affects the production of traction forces.

### 3.5. c-Abl Kinase Enhances Gal3 Secretion and Production of Traction Force

**Figure 6:**
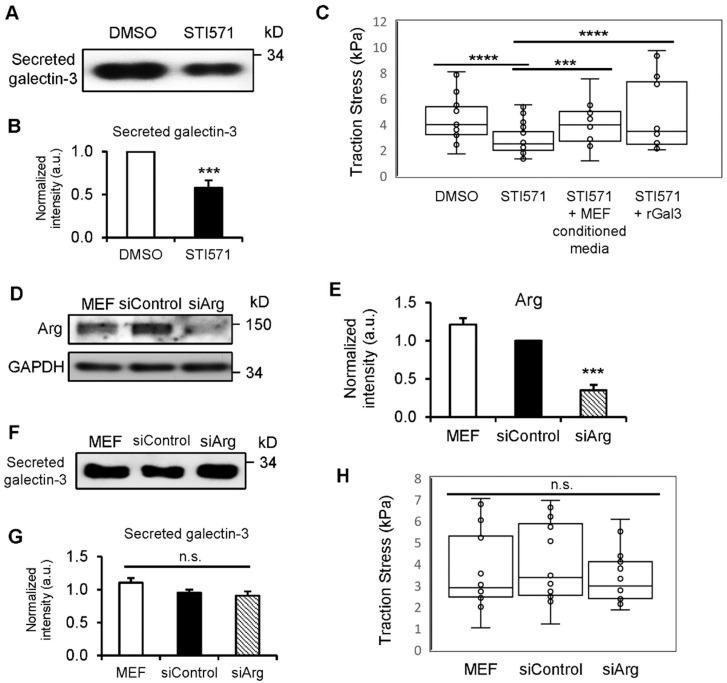
c-Abl kinase, but not Arg kinase, regulates Gal3 secretion and traction force production, which is recovered by extracellular Gal3. (**A**) Western blot of Gal3 from conditioned media of MEF cells treated with either vehicle (DMSO) or Abl kinase inhibitor (STI571). (**B**) Quantification of secreted Gal3 levels of MEF cells with DMSO or STI571. Normalized intensity expressed in arbitrary units in the bar graphs is an average of three independent experiments. (**C**) Bar graph of average traction stress exerted by MEF cells treated with either DMSO (n = 19) or STI571 (n = 37), and MEF cells treated with STI571 with conditioned media from wild-type MEF cells (STI571 + MEF conditioned media; n = 15) or with rGal3 (STI571 + rGal3; n = 18) exogenously added to the media. (**D**) Western blot of Arg from cell lysates of wild-type MEF cells (MEF) and cells nucleofected with control siRNA (siControl) or Arg siRNA (siArg). GAPDH served as the loading control. (**E**) Quantification of Arg levels of MEF cells and cells with siControl or siArg. Normalized intensity expressed in arbitrary units in the bar graphs is an average of three independent experiments. (**F**) Western blot of Gal3 from conditioned media of MEF cells and cells treated with siControl or siArg. (**G**) Quantification of secreted Gal3 levels of MEF cells and cells with siControl or siArg. Normalized intensity expressed in arbitrary units in the bar graphs is an average of three independent experiments. Total protein level by Coomassie Blue staining is used to normalize the intensity of secreted Gal3. (**H**) Bar graph of average traction stress exerted by MEF cells (n = 14), and cells treated with siControl (n = 22) or siArg (n = 21). Error bars represent mean ± SEM. Student’s *t*-test *** *p* < 0.005; **** *p* < 0.001; n.s., not significant.

Previous studies have found that Gal3 is phosphorylated by c-Abl kinase on three tyrosine residues: Y79, Y107, and Y117 [21,22]. To determine whether c-Abl kinase-mediated tyrosine phosphorylation of Gal3 influences its secretion, we compared Gal3 levels in conditioned media from MEF cells treated with either vehicle (DMSO) or an Abl kinase inhibitor (STI571). When the kinase activity was inhibited, the level of Gal3 secretion decreased remarkably (Figure 6A,B). We further performed TFM to examine if the decreased Gal3 secretion by c-Abl kinase also affects traction force. STI571-treated MEF cells had significantly lower traction compared to that of DMSO-treated cells (Figure 6C). The decreased traction in STI571-treated MEF cells was rescued by adding conditioned media from wild-type MEF cells, which contain secreted Gal3, as we have previously reported [16], or by exogenously adding rGal3 (Figure 6C). This suggests that the defect in traction observed in STI571-treated cells is caused by reduced extracellular Gal3 levels. STI571 is also known to inhibit the Abelson-related gene (Arg, also known as Abl2), which is within the Abl family of non-receptor tyrosine kinases [64]. To examine if the decreased Gal3 secretion and traction in STI571-treated MEF cells could be mediated by Arg, we knocked down Arg expression using siRNA and performed Western blot and TFM (Figure 6D,E,H). However, there was no significant difference in the secretion of Gal3 (Figure 6F,G) or traction force generation (Figure 6H) from Arg siRNA-treated MEF cells compared to cells treated with control siRNA. Together, this suggests that the c-Abl-mediated tyrosine phosphorylation of Gal3 affects its secretion and the generation of traction force mediated by extracellular Gal3.

### 3.6. Y107 Phosphorylation of Gal3 Is Critical for Its Secretion and the Generation of Traction Forces

**Figure 7:**
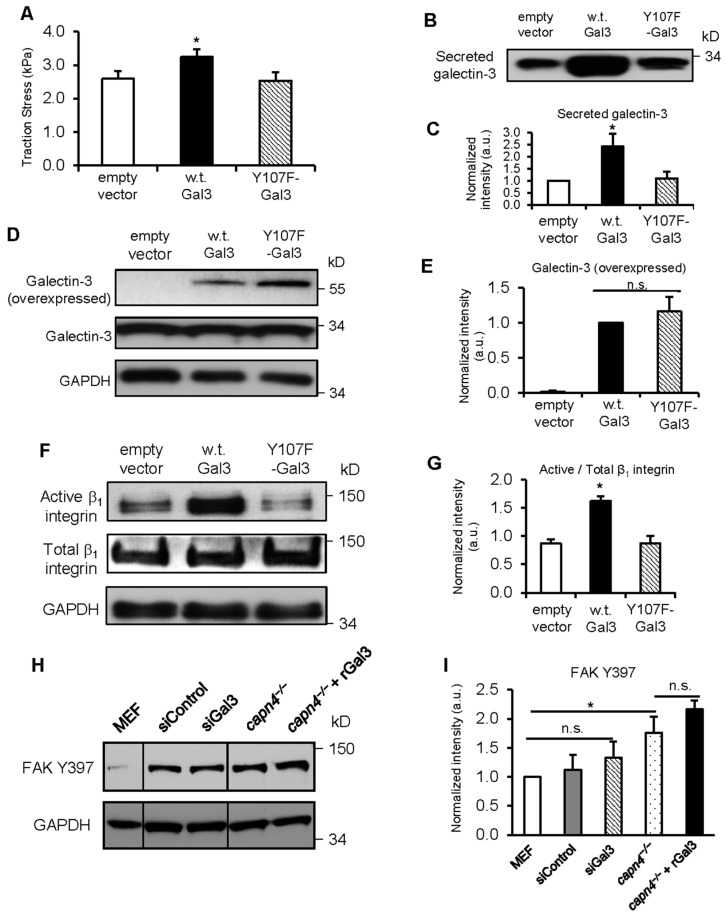
Capn4-mediated Y107 phosphorylation of Gal3 is required for its secretion and subsequent generation of traction forces (**A**) Bar graph of average traction stress exerted by MEF cells nucleofected with pEGFP-N3 (empty vector; n = 25), pECFP-Gal3 (w.t. Gal3; n = 19), or pEGFP-Y107F-Gal3 (Y107F-Gal3; n = 25). (**B**) Western blot of Gal3 from conditioned media of MEF cells with empty vector, w.t. Gal3 or Y107F-Gal3. (**C**) Quantification of secreted Gal3 levels of MEF cells with empty vector, w.t. Gal3 or Y107F-Gal3. Total protein level by Coomassie Blue staining is used to normalize the intensity of secreted Gal3 (**D**) Western blot of overexpressed and endogenous Gal3 from cell lysates of empty vector, w.t. Gal3 or Y107F-Gal3 in MEF cells. GAPDH served as the loading control. (**E**) Quantification of overexpressed Gal3 levels of MEF cells with empty vector, w.t. Gal3 or Y107F-Gal3. Normalized intensity expressed in arbitrary units in the bar graphs is an average of three independent experiments. Error bars represent mean ± SEM. Student’s *t*-test * *p* < 0.05; n.s., not significant. Extracellular Gal3 does not alter FAK autophosphorylation. (**F**) Western blot of active and total β1 integrin from cell lysates of MEF cells with empty vector, w.t. Gal3 or Y107F-Gal3. GAPDH served as the loading control. (**G**) Quantification of active β1 integrin levels of MEF cells with empty vector, w.t. Gal3 or Y107F-Gal3. (**H**) Western blot of FAK Y397 phosphorylation from cell lysates of w.t. MEF cells, cells nucleofected with siControl or siGal3, *capn4^−/−^* MEF cells, and *capn4^−/−^* MEF cells with recombinant Gal3 added exogenously. GAPDH served as the loading control. (**I**) Quantification of FAK Y397 phosphorylation levels of MEF cells in C. Normalized intensity expressed in arbitrary units in the bar graphs is an average of three independent experiments. Error bars represent mean ± SEM. Student’s *t*-test * *p* < 0.05; n.s., not significant.

It was previously determined that Y107 of Gal3 is the primary phosphorylation target of c-Abl kinase and is significant in the morphology and motility of breast cancer cells [21]. We next evaluated whether the Y107 phosphorylation of Gal3 is crucial for the secretion of Gal3 and to produce traction forces. MEF cells were overexpressed with an empty vector, wild-type Gal3 (w.t. Gal3) or a non-phosphorylatable mutant form of Gal3 at Y107; tyrosine-107 residue has been substituted with phenylalanine (Y107F-Gal3). We compared the magnitudes of traction force among the three cell lines and found that w.t. Gal3 but not Y107F-Gal3 overexpression in MEF cells resulted in greater traction being generated than that of control cells (Figure 7A). This is consistent with the observation that conditioned media showed no significant increase in extracellular Gal3 when Y107F-Gal3 was overexpressed, whereas more than a two-fold increase in Gal3 secretion occurred with w.t. Gal3 overexpression (Figure 7B,C). Intracellular Gal3 overexpression levels were similar between the cells with w.t. Gal3 or Y107F-Gal3, and endogenous Gal3 levels remained similar among all three cell lines (Figure 7D,E). These results reveal the importance of the phosphorylation of Gal3 at Y107 for its secretion to promote traction force generation. In addition, we applied those three plasmid constructs (empty vector, w.t. Gal3 and Y107F-Gal3) in *capn4^−/−^* MEF cells to determine if overexpressing Gal3 has any effect on traction or secretion of Gal3 in *capn4^−/−^* MEF cells (Figure S3A–D). As expected, overexpressing w.t. Gal3 or Y107F-Gal3 could not restore reduced traction (Figure S3A) or Gal3 secretion (Figure S3B) previously observed in *capn4^−/−^* MEF cells while overexpressed and endogenous Gal3 levels were similar between w.t. Gal3 and Y107F-Gal3 cells (Figure S3C,D). These results strengthen our hypothesis that Capn4 acts upstream of Gal3 in this signaling pathway to generate traction forces and that Capn4-mediated phosphorylation of Gal3 is the key step in the route of Gal3 secretion.

### 3.7. Active Integrin β1 May Participate in Extracellular Gal3-Mediated Traction Force Production, Possibly through a FAK-Independent Mechanism

Previous studies have implicated Gal3 in the clustering of integrin receptors [32,33]. Given that all the experiments used in this study have involved surface coating of fibronectin, we reasoned that Gal3 could be working through a fibronectin receptor to modulate traction force, adhesion localization, and strengthening. Integrins that serve as the receptors for fibronectin include α5β1, α4β1, αIIβ3, and αVβ3 [65]. Specifically, α5β1 clustering is required for the formation of strong fibronectin-bound adhesions [66]. Since overexpression of wild-type Gal3 leads to an increase in its secretion and traction force generation, we asked if the increased traction mediated by Gal3 could be due to the activation of β1 integrin. We measured the levels of active and total β1 integrin in protein extracts from MEF cells containing an empty vector, w.t. Gal3 or Y107F-Gal3, all grown on fibronectin-coated surfaces. We found higher active β1 integrin levels when w.t. Gal3 was overexpressed than that of the empty vector, whereas almost no significant change was detected upon Y107F-Gal3 overexpression (Figure 7F,G). This suggests that the extracellular Gal3-mediated increase in traction is modulated through β 1 integrin.

Tyrosine phosphorylation of focal adhesion kinase (FAK) residue Y397 occurs through autophosphorylation [67]. It is the initial step in the activation of FAK leading to the phosphorylation of numerous other FAK tyrosine residues. Tyrosine phosphorylation of FAK is associated with cell migration including mechanosensing and traction force [68,69,70,71]. To determine if Gal3-mediated regulation of traction force, focal adhesion turnover, and adhesion strength is mediated through the autophosphorylation of FAK, we checked the levels of Y397 phosphorylated FAK present in wild-type MEF cells and *capn4^−/−^* MEF cells. Levels of Y397 in *capn4^−/−^* MEF cells are approximately two-fold higher than levels in wild-type MEF cells (Figure 7H,I). The addition of rGal3 to *capn4^−/−^* MEF cells did not change the FAK Y397 phosphorylation compared to levels in untreated *capn4^−/−^* MEF cells. The silencing of Gal3 in MEF cells did not alter the levels of Y397 FAK phosphorylation. This result suggests that Gal3-mediated regulation of cell migration is not modulated through the FAK pathway.

## 4. Discussion

Cell migration is a process that is influenced by a myriad of intracellular and extracellular factors that may be biochemical or biophysical. It is carefully coordinated by multiple signal transduction pathways, many of which are not fully understood. Our group had previously shown the importance of the calcium-dependent family of proteases, namely calpains, in the regulation of two biophysical parameters: traction force and mechanosensing [15]. In addition, it was found that focal adhesion dynamics and strengthening were also affected by the calpain family. We concluded that Capn4 functions independently of the proteolytic activity of the large catalytic subunits in the process of generating traction force. The large subunits, calpains 1 and 2, along with the small subunit are involved in sensing global stiffness and locally applied transient mechanical stimulations. Thus, the calpains have provided a means to separate spatially and temporally, traction force and mechanosensing.

There are several different ways of measuring cellular traction, and the mechanism used to generate traction force is being investigated by a number of groups [72,73]. To further understand the function of calpains in traction force generation and mechanosensing, we looked for differential tyrosine phosphorylation levels in the absence of each of the calpain subunits. We found that in the absence of Capn4, the protein Gal3 was no longer phosphorylated. We also discovered that this lack of tyrosine phosphorylation prevented its secretion [16].

Gal3 has been known to function in cell migration and ECM remodeling. Many studies suggest that it modulates ECM interactions from the outside of the cell, both under normal conditions and in cancer cells [29,33]. Thus, the fact that Gal3 secretion was mediated by tyrosine phosphorylation, which is indirectly regulated by Capn4, prompted us to look at its influence on traction force when exogenously added to Capn4-deficient cells. Our results obtained upon the addition of recombinant Gal3 to the culture medium concur with studies by other groups suggesting that the protein has an extracellular function. We were able to rescue defects in traction force that occur in Capn4-deficient cells (Figure 1). The addition of recombinant Gal3 also mediated changes in the size of adhesions from small contacts (adhesions that were less than 0.5 µm^2^) to mature adhesions (>1.5 µm^2^) and helped strengthen the cell adherence (Figure 2). It has previously been shown that forces are greatest at the leading edge of migrating cells and that smaller adhesions generate greater forces [74]; however, the *capn4^−/−^* cells despite having numerous smaller adhesions have weak traction but also have abnormal actin organization. The addition of recombinant Gal3 resulted in fewer small contacts as compared to Capn4-deficient fibroblasts that had a significantly greater number of focal complexes.

The focal adhesion lengths diversified upon the addition of recombinant Gal3, and the adhesion localization defect was resolved. Therefore, the rescue of the *capn4^−/−^* adhesion size and localization defect could also explain the rebalance in adhesion strength when extracellular Gal3 was added. Together these results support the popular idea that Gal3 in the extracellular environment forms a lattice, which then helps cluster and activate integrins [32,33]. Once integrins are activated, they trigger numerous intracellular signal transduction pathways that can lead to increased adhesion maturation, improved strength, and greater forces. The primary fibronectin receptor α5β1 provides a reasonable target as its function in strengthening cell adhesion and migration is well-documented [66]. We found an increase in active β1 integrin level when w.t. Gal3 was overexpressed although Y107F-Gal3 overexpression did not make any notable change. As w.t. Gal3 overexpression resulted in greater Gal3 secretion and traction, the increased β1 integrin activation with w.t. Gal3 overexpression implies the link between extracellular Gal3-mediated traction and the activation of β1 integrin (Figure 7). Moving further downstream from β1 integrin activation is FAK autophosphorylation at the tyrosine 397 residue. Upon Gal3 addition, we did not observe a dramatic change in the already elevated levels of Y397 phosphorylation present in Capn4-deficient cells (Figure 7). The simplest explanation for our data is that extracellular Gal3 activates pathways that require β1 integrin activation but are not followed by FAK Y397 phosphorylation. Instead, as proposed in other literature, it may transduce through Src kinase activation independent of FAK autophosphorylation [75]. This would identify a previously unknown mechanotransduction pathway that signals only for the production of traction forces and not mechanosensing.

As with previous studies that have shown slower migration rates for *capn4^−/−^* cells on fibronectin-coated surfaces as compared to wild-type cells [42], we see that the rate of migration of *capn4^−/−^* cells is not significantly different from wild-type fibroblasts (Figure 3A). This difference could have been due to the concentration of fibronectin (5 μg/cm^2^) used in our studies that is higher than those used previously for these migration studies. Previous research has also shown that high concentrations of fibronectin reduce the rate of migration by modulating Rho GTPases through integrins [76]. Nonetheless, the increase in linear speed observed when recombinant Gal3 is added exogenously to Capn4-deficient fibroblast supports the notion that Gal3 has been shown to cluster and activate integrins. Gal3 is also proposed to form a lattice that promotes fibrillogenesis, thus providing another potential route to modulate the rate and direction of migration [34].

It has been proposed that microtubule depolymerization-induced traction force regulation can be mediated by two distinct pathways: a myosin-II-dependent/FAK-independent pathway and a FAK-regulated/myosin-II-independent pathway [77]. Because when extracellular Gal3 is added to *capn4^−/−^* cells we see an increase in traction without a correspondingly significant increase in the levels of FAK Y397 phosphorylation (Figure 1 and 7), it is plausible that Gal3/Capn4-mediated regulation of force occurs via the myosin-II dependent pathway. Furthermore, we found that the FAK Y397 phosphorylation levels are higher in *capn4^−/−^* cells as compared to wild-type MEF cells, but the levels of traction force produced are inversely correlated. An alternative, but the speculative mechanism is that Capn4 could bind to a phosphatase-interacting protein, such as PSTPIP1 through its SH3 domain. Previous studies have shown that Capn4 binds to proteins through SH3 domains present in the interacting partner [78]. Similarly, PSTPIP1 also binds to its partners through SH3 domains present in its structure [79,80]. PSTPIP1 has been shown to direct PEST-type protein tyrosine phosphatase (PTP) to c-Abl kinase [81]. If it interacts with Capn4, it will prevent a PSTPIP1–PEST PTP interaction, thus preventing the delivery of PEST-type protein tyrosine phosphatase to the c-Abl kinase. Thus, c-Abl kinase remains active, phosphorylating its substrate Gal3.

**Figure 8:**
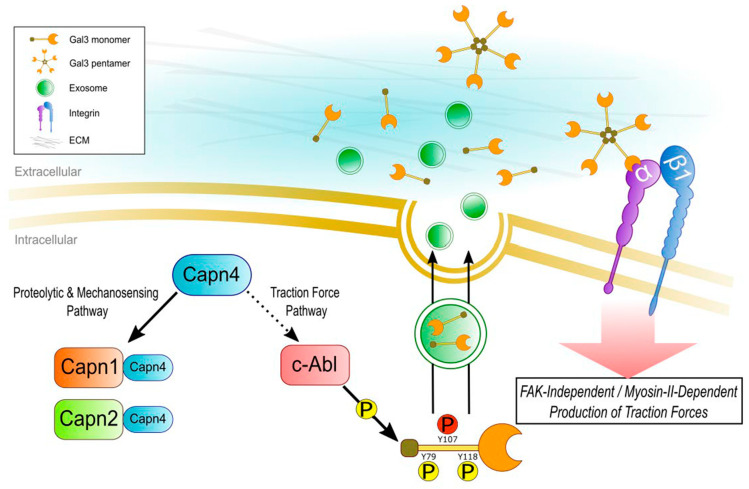
Postulated pathway for Capn4-triggered Gal3 phosphorylation, secretion, and consequent traction force production. All direct interactions, phosphorylation, secretion, and clustering have been formulated based on experimental or published results. Capn4, together with large catalytic subunits of Capn1 and 2 holoenzymes, modulates proteolytic and mechanosensing pathways. On the other hand, Capn4 governs the traction force pathway, independently from large subunits. Capn4 may help in stimulating c-Abl kinase activation resulting in tyrosine phosphorylation of Gal3. c-Abl kinase phosphorylates Gal3 on three tyrosine residues: Y79, Y107, and Y118. Tyrosine-phosphorylated Gal3, on Y107, could be secreted through exosomes or by other non-classical pathways. Secreted Gal3 can form oligomers through its N-terminal domain in the ECM and can link the cell to the ECM through clustering and/or activating cell surface receptors including integrins. As a result, active β1 integrin-signaling may occur in an FAK-independent, myosin-II-mediated manner to produce traction forces during cell migration.

Based on our experimental data and previously published information, we have proposed a Capn4- and extracellular Gal3-mediated traction force mechanism (Figure 8). We have demonstrated that Capn4 not only participates in the mechanosensing pathway in conjunction with Capn1 and 2 catalytic subunits as a form of holoenzyme but also triggers a traction force pathway through Gal3 phosphorylation and secretion independently from catalytic subunits. Three tyrosine residues of Gal3, mainly on Y107, are phosphorylated by c-Abl kinase leading to the secretion of phosphorylated Gal3 (Figure 6 and Figure 7) through exosomes or other non-classical secretion pathways [35,36,37,38,39]. Once secreted, Gal3 forms oligomers through its N-terminal domain in the extracellular space and causes fibrillogenesis followed by integrin clustering and activation and then mechanotransduction [19,34]. This is then translated into force generation via a myosin-II-mediated, FAK-independent pathway [77]. As the addition of Gal3 was unable to rescue *capn4^−/−^* defects in both global and applied mechanosensing, we have further strengthened our previous observation that Capn4 functions independently of the large subunits in the production of traction forces. Furthermore, we can mechanistically separate, through the secretion of Gal3, a pathway independent of the mechanosensing pathway. Finally, we would like to suggest that this mechanism is potentially involved in advancing the fibrotic diseases to which galectin-3 and its extracellular functions have already been linked. The results of our study support the need for further exploration of Gal3 as a biomedical target for several diseases in which it has been implicated.

## Data Availability

The original contributions presented in the study are included in the article/Supplementary Material, further inquiries can be directed to the corresponding author/s.

## Supplementary Materials

The following supporting information can be downloaded at: https://www.mdpi.com/article/10.3390/biomedicines12061247/s1, Figure S1. Representative vector plot depicts the magnitude and direction of traction stress exerted by a wild-type MEF cell, The vectors indicate the direction and magnitude of traction stress. Figure S2. Speed and persistence on Polyacrylamide substrates. (A) Linear speed of wild-type and *capn4^−/−^* MEF cells on fibronectin-coated polyacrylamide substrates. (B) Persistence of wild- type (n = 15) and *capn4^−/−^* MEF cells (n = 15) on fibronectin-coated polyacrylamide substrates. Each cell was imaged 2 hours, each trial 2 cells of each cell type was observed, for a total of 7 trials. Error bars represent mean ± SEM. Figure S3. (A) Bar graph of average traction stress exerted by *capn4^−/−^* MEF cells nucleofected with pEGFP-N3 (empty vector; n = 19), pECFP-Gal3 (w.t. Gal3; n = 15) or pEGFP-Y107F-Gal3 (Y107F-Gal3; n = 14). (B) Western blot of Gal3 from conditioned media of *capn4^−/−^* MEF cells with empty vector, w.t. Gal3 or Y107F-Gal3. Quantification of data are not measurable for a graph. (C) Western blot of overexpressed and endogenous Gal3 from cell lysates of empty vector, w.t. Gal3 or Y107F-Gal3 in *capn4^−/−^* MEF cells. GAPDH served as the loading control. (D) Quantification of overexpressed Gal3 levels of *capn4^−/−^* MEF cells with empty vector, w.t. Gal3 or Y107F-Gal3. Normalized intensity expressed in arbitrary units in the bar graphs is an average of three independent experiments. Error bars represent mean ± SEM. n.s., not significant.

## References

[1] Huttenlocher A., Sandborg R.R., Horwitz A.F. (1995). Adhesion in cell migration. Curr. Opin. Cell Biol..

[2] Li S., Guan J.L., Chien S. (2005). Biochemistry and biomechanics of cell motility. Annu. Rev. Biomed. Eng..

[3] Gardel M.L., Schneider I.C., Aratyn-Schaus Y., Waterman C.M. (2010). Mechanical integration of actin and adhesion dynamics in cell migration. Annu. Rev. Cell Dev. Biol..

[4] Pandya P., Orgaz J.L., Sanz-Moreno V. (2017). Actomyosin contractility and collective migration: May the force be with you. Curr. Opin. Cell Biol..

[5] De R., Zemel A., Safran S.A. (2010). Theoretical concepts and models of cellular mechanosensing. Methods Cell Biol..

[6] Vogel V., Sheetz M. (2006). Local force and geometry sensing regulate cell functions. Nat. Rev. Mol. Cell Biol..

[7] Jansen K.A., Atherton P., Ballestrem C. (2017). Mechanotransduction at the cell-matrix interface. Semin. Cell Dev. Biol..

[8] Fouchard J., Mitrossilis D., Asnacios A. (2011). Acto-myosin based response to stiffness and rigidity sensing. Cell Adh. Migr..

[9] Prager-Khoutorsky M., Lichtenstein A., Krishnan R., Rajendran K., Mayo A., Kam Z., Geiger B., Bershadsky A.D. (2011). Fibroblast polarization is a matrix-rigidity-dependent process controlled by focal adhesion mechanosensing. Nat. Cell Biol..

[10] Weng S., Fu J. (2011). Synergistic regulation of cell function by matrix rigidity and adhesive pattern. Biomaterials.

[11] Trichet L., Le Digabel J., Hawkins R.J., Vedula S.R., Gupta M., Ribrault C., Hersen P., Voituriez R., Ladoux B. (2012). Evidence of a large-scale mechanosensing mechanism for cellular adaptation to substrate stiffness. Proc. Natl. Acad. Sci. USA.

[12] Wang Y.L. (2009). Traction forces and rigidity sensing of adherent cells. Conf. Proc. IEEE Eng. Med. Biol. Soc..

[13] Califano J.P., Reinhart-King C.A. (2010). Substrate Stiffness and Cell Area Predict Cellular Traction Stresses in Single Cells and Cells in Contact. Cell Mol. Bioeng..

[14] Chan C.E., Odde D.J. (2008). Traction dynamics of filopodia on compliant substrates. Science.

[15] Undyala V.V., Dembo M., Cembrola K., Perrin B.J., Huttenlocher A., Elce J.S., Greer P.A., Wang Y.L., Beningo K.A. (2008). The calpain small subunit regulates cell-substrate mechanical interactions during fibroblast migration. J. Cell Sci..

[16] Menon S., Kang C.M., Beningo K.A. (2011). Galectin-3 secretion and tyrosine phosphorylation is dependent on the calpain small subunit, Calpain 4. Biochem. Biophys. Res. Commun..

[17] Krzeslak A., Lipinska A. (2004). Galectin-3 as a multifunctional protein. Cell Mol. Biol. Lett..

[18] Nakahara S., Raz A. (2006). On the role of galectins in signal transduction. Methods Enzymol..

[19] Ahmad N., Gabius H.J., Andre S., Kaltner H., Sabesan S., Roy R., Liu B., Macaluso F., Brewer C.F. (2004). Galectin-3 precipitates as a pentamer with synthetic multivalent carbohydrates and forms heterogeneous cross-linked complexes. J. Biol. Chem..

[20] Houzelstein D., Goncalves I.R., Fadden A.J., Sidhu S.S., Cooper D.N., Drickamer K., Leffler H., Poirier F. (2004). Phylogenetic analysis of the vertebrate galectin family. Mol. Biol. Evol..

[21] Balan V., Nangia-Makker P., Jung Y.S., Wang Y., Raz A. (2010). Galectin-3: A novel substrate for c-Abl kinase. Biochim. Biophys. Acta.

[22] Li X., Ma Q., Wang J., Liu X., Yang Y., Zhao H., Wang Y., Jin Y., Zeng J., Li J. (2010). c-Abl and Arg tyrosine kinases regulate lysosomal degradation of the oncoprotein Galectin-3. Cell Death Differ..

[23] Dumic J., Dabelic S., Flogel M. (2006). Galectin-3: An open-ended story. Biochim. Biophys. Acta.

[24] Haudek K.C., Spronk K.J., Voss P.G., Patterson R.J., Wang J.L., Arnoys E.J. (2010). Dynamics of galectin-3 in the nucleus and cytoplasm. Biochim. Biophys. Acta.

[25] Liu F.T., Patterson R.J., Wang J.L. (2002). Intracellular functions of galectins. Biochim. Biophys. Acta.

[26] Dange M.C., Agarwal A.K., Kalraiya R.D. (2015). Extracellular galectin-3 induces MMP9 expression by activating p38 MAPK pathway via lysosome-associated membrane protein-1 (LAMP1). Mol. Cell Biochem..

[27] Mori Y., Akita K., Yashiro M., Sawada T., Hirakawa K., Murata T., Nakada H. (2015). Binding of Galectin-3, a beta-Galactoside-binding Lectin, to MUC1 Protein Enhances Phosphorylation of Extracellular Signal-regulated Kinase 1/2 (ERK1/2) and Akt, Promoting Tumor Cell Malignancy. J. Biol. Chem..

[28] Colomb F., Wang W., Simpson D., Zafar M., Beynon R., Rhodes J.M., Yu L.G. (2017). Galectin-3 interacts with the cell-surface glycoprotein CD146 (MCAM, MUC18) and induces secretion of metastasis-promoting cytokines from vascular endothelial cells. J. Biol. Chem..

[29] Ochieng J., Furtak V., Lukyanov P. (2004). Extracellular functions of galectin-3. Glycoconj. J..

[30] Boscher C., Nabi I.R. (2013). Galectin-3- and phospho-caveolin-1-dependent outside-in integrin signaling mediates the EGF motogenic response in mammary cancer cells. Mol. Biol. Cell.

[31] Melo F.H., Butera D., Junqueira Mde S., Hsu D.K., da Silva A.M., Liu F.T., Santos M.F., Chammas R. (2011). The promigratory activity of the matricellular protein galectin-3 depends on the activation of PI-3 kinase. PLoS ONE.

[32] Yang E.H., Rode J., Howlader M.A., Eckermann M., Santos J.T., Hernandez Armada D., Zheng R., Zou C., Cairo C.W. (2017). Galectin-3 alters the lateral mobility and clustering of beta1-integrin receptors. PLoS ONE.

[33] Goetz J.G., Joshi B., Lajoie P., Strugnell S.S., Scudamore T., Kojic L.D., Nabi I.R. (2008). Concerted regulation of focal adhesion dynamics by galectin-3 and tyrosine-phosphorylated caveolin-1. J. Cell Biol..

[34] Lagana A., Goetz J.G., Cheung P., Raz A., Dennis J.W., Nabi I.R. (2006). Galectin binding to Mgat5-modified N-glycans regulates fibronectin matrix remodeling in tumor cells. Mol. Cell Biol..

[35] Gong H.C., Honjo Y., Nangia-Makker P., Hogan V., Mazurak N., Bresalier R.S., Raz A. (1999). The NH2 terminus of galectin-3 governs cellular compartmentalization and functions in cancer cells. Cancer Res..

[36] Lindstedt R., Apodaca G., Barondes S.H., Mostov K.E., Leffler H. (1993). Apical secretion of a cytosolic protein by Madin-Darby canine kidney cells. Evidence for polarized release of an endogenous lectin by a nonclassical secretory pathway. J. Biol. Chem..

[37] Sato S., Burdett I., Hughes R.C. (1993). Secretion of the baby hamster kidney 30-kDa galactose-binding lectin from polarized and nonpolarized cells: A pathway independent of the endoplasmic reticulum-Golgi complex. Exp. Cell Res..

[38] Zhu W.Q., Ochieng J. (2001). Rapid release of intracellular galectin-3 from breast carcinoma cells by fetuin. Cancer Res..

[39] Banfer S., Schneider D., Dewes J., Strauss M.T., Freibert S.A., Heimerl T., Maier U.G., Elsasser H.P., Jungmann R., Jacob R. (2018). Molecular mechanism to recruit galectin-3 into multivesicular bodies for polarized exosomal secretion. Proc. Natl. Acad. Sci. USA.

[40] Papusheva E., Heisenberg C.P. (2010). Spatial organization of adhesion: Force-dependent regulation and function in tissue morphogenesis. EMBO J..

[41] Wolfenson H., Henis Y.I., Geiger B., Bershadsky A.D. (2009). The heel and toe of the cell’s foot: A multifaceted approach for understanding the structure and dynamics of focal adhesions. Cell Motil. Cytoskeleton.

[42] Dourdin N., Bhatt A.K., Dutt P., Greer P.A., Arthur J.S., Elce J.S., Huttenlocher A. (2001). Reduced cell migration and disruption of the actin cytoskeleton in calpain-deficient embryonic fibroblasts. J. Biol. Chem..

[43] Arthur J.S., Elce J.S., Hegadorn C., Williams K., Greer P.A. (2000). Disruption of the murine calpain small subunit gene, Capn4: Calpain is essential for embryonic development but not for cell growth and division. Mol. Cell Biol..

[44] Franco S., Perrin B., Huttenlocher A. (2004). Isoform specific function of calpain 2 in regulating membrane protrusion. Exp. Cell Res..

[45] Lo C.M., Wang H.B., Dembo M., Wang Y.L. (2000). Cell movement is guided by the rigidity of the substrate. Biophys. J..

[46] Beningo K.A., Lo C.M., Wang Y.L. (2002). Flexible polyacrylamide substrata for the analysis of mechanical interactions at cell-substratum adhesions. Methods Cell Biol..

[47] Dembo M., Wang Y.L. (1999). Stresses at the cell-to-substrate interface during locomotion of fibroblasts. Biophys. J..

[48] Marganski W.A., Dembo M., Wang Y.L. (2003). Measurements of cell-generated deformations on flexible substrata using correlation-based optical flow. Methods Enzymol..

[49] Guo W.H., Frey M.T., Burnham N.A., Wang Y.L. (2006). Substrate rigidity regulates the formation and maintenance of tissues. Biophys. J..

[50] Ilina O., Friedl P. (2009). Mechanisms of collective cell migration at a glance. J. Cell Sci..

[51] Lauffenburger D.A., Horwitz A.F. (1996). Cell migration: A physically integrated molecular process. Cell.

[52] Petrie R.J., Doyle A.D., Yamada K.M. (2009). Random versus directionally persistent cell migration. Nat. Rev. Mol. Cell Biol..

[53] Munevar S., Wang Y.L., Dembo M. (2001). Distinct roles of frontal and rear cell-substrate adhesions in fibroblast migration. Mol. Biol. Cell.

[54] Freund J.B., Goetz J.G., Hill K.L., Vermot J. (2012). Fluid flows and forces in development: Functions, features and biophysical principles. Development.

[55] Guilak F., Cohen D.M., Estes B.T., Gimble J.M., Liedtke W., Chen C.S. (2009). Control of stem cell fate by physical interactions with the extracellular matrix. Cell Stem Cell.

[56] Menon S., Beningo K.A. (2011). Cancer cell invasion is enhanced by applied mechanical stimulation. PLoS ONE.

[57] Discher D.E., Janmey P., Wang Y.L. (2005). Tissue cells feel and respond to the stiffness of their substrate. Science.

[58] Discher D.E., Mooney D.J., Zandstra P.W. (2009). Growth factors, matrices, and forces combine and control stem cells. Science.

[59] Engler A.J., Sen S., Sweeney H.L., Discher D.E. (2006). Matrix elasticity directs stem cell lineage specification. Cell.

[60] Pelham R.J., Wang Y. (1997). Cell locomotion and focal adhesions are regulated by substrate flexibility. Proc. Natl. Acad. Sci. USA.

[61] Pittenger M.F., Mackay A.M., Beck S.C., Jaiswal R.K., Douglas R., Mosca J.D., Moorman M.A., Simonetti D.W., Craig S., Marshak D.R. (1999). Multilineage potential of adult human mesenchymal stem cells. Science.

[62] Flanagan L.A., Ju Y.E., Marg B., Osterfield M., Janmey P.A. (2002). Neurite branching on deformable substrates. Neuroreport.

[63] Saha K., Keung A.J., Irwin E.F., Li Y., Little L., Schaffer D.V., Healy K.E. (2008). Substrate modulus directs neural stem cell behavior. Biophys. J..

[64] Hantschel O., Rix U., Superti-Furga G. (2008). Target spectrum of the BCR-ABL inhibitors imatinib, nilotinib and dasatinib. Leuk. Lymphoma.

[65] Plow E.F., Haas T.A., Zhang L., Loftus J., Smith J.W. (2000). Ligand binding to integrins. J. Biol. Chem..

[66] Roca-Cusachs P., Gauthier N.C., Del Rio A., Sheetz M.P. (2009). Clustering of alpha(5)beta(1) integrins determines adhesion strength whereas alpha(v)beta(3) and talin enable mechanotransduction. Proc. Natl. Acad. Sci. USA.

[67] Mitra S.K., Hanson D.A., Schlaepfer D.D. (2005). Focal adhesion kinase: In command and control of cell motility. Nat. Rev. Mol. Cell Biol..

[68] Michael K.E., Dumbauld D.W., Burns K.L., Hanks S.K., Garcia A.J. (2009). Focal adhesion kinase modulates cell adhesion strengthening via integrin activation. Mol. Biol. Cell.

[69] Pirone D.M., Liu W.F., Ruiz S.A., Gao L., Raghavan S., Lemmon C.A., Romer L.H., Chen C.S. (2006). An inhibitory role for FAK in regulating proliferation: A link between limited adhesion and RhoA-ROCK signaling. J. Cell Biol..

[70] Schober M., Raghavan S., Nikolova M., Polak L., Pasolli H.A., Beggs H.E., Reichardt L.F., Fuchs E. (2007). Focal adhesion kinase modulates tension signaling to control actin and focal adhesion dynamics. J. Cell Biol..

[71] Wang H.B., Dembo M., Hanks S.K., Wang Y. (2001). Focal adhesion kinase is involved in mechanosensing during fibroblast migration. Proc. Natl. Acad. Sci. USA.

[72] Kraning-Rush C.M., Carey S.P., Califano J.P., Reinhart-King C.A. (2012). Quantifying traction stresses in adherent cells. Methods Cell Biol..

[73] Wang J.H., Lin J.S. (2007). Cell traction force and measurement methods. Biomech. Model. Mechanobiol..

[74] Beningo K.A., Dembo M., Kaverina I., Small J.V., Wang Y.L. (2001). Nascent focal adhesions are responsible for the generation of strong propulsive forces in migrating fibroblasts. J. Cell Biol..

[75] Arias-Salgado E.G., Lizano S., Sarkar S., Brugge J.S., Ginsberg M.H., Shattil S.J. (2003). Src kinase activation by direct interaction with the integrin beta cytoplasmic domain. Proc. Natl. Acad. Sci. USA.

[76] Cox E.A., Sastry S.K., Huttenlocher A. (2001). Integrin-mediated adhesion regulates cell polarity and membrane protrusion through the Rho family of GTPases. Mol. Biol. Cell.

[77] Rape A., Guo W.H., Wang Y.L. (2011). Microtubule depolymerization induces traction force increase through two distinct pathways. J. Cell Sci..

[78] Rosenberger G., Gal A., Kutsche K. (2005). AlphaPIX associates with calpain 4, the small subunit of calpain, and has a dual role in integrin-mediated cell spreading. J. Biol. Chem..

[79] Wu Y., Spencer S.D., Lasky L.A. (1998). Tyrosine phosphorylation regulates the SH3-mediated binding of the Wiskott-Aldrich syndrome protein to PSTPIP, a cytoskeletal-associated protein. J. Biol. Chem..

[80] Baum W., Kirkin V., Fernandez S.B., Pick R., Lettau M., Janssen O., Zornig M. (2005). Binding of the intracellular Fas ligand (FasL) domain to the adaptor protein PSTPIP results in a cytoplasmic localization of FasL. J. Biol. Chem..

[81] Cong F., Spencer S., Cote J.F., Wu Y., Tremblay M.L., Lasky L.A., Goff S.P. (2000). Cytoskeletal protein PSTPIP1 directs the PEST-type protein tyrosine phosphatase to the c-Abl kinase to mediate Abl dephosphorylation. Mol. Cell.

