# Supplementary material for: Calpain Small Subunit Mediated Secretion of Galectin-3 Regulates Traction Stress
Source: Biomedicines. 2024 Jun 4;12(6):1247. doi: 10.3390/biomedicines12061247 (PMC11200796; doi:10.3390/biomedicines12061247)
Supplement: Supplementary file 1 [file biomedicines-12-01247-s001.zip › biomedicines-3020236-supplementary.pdf]

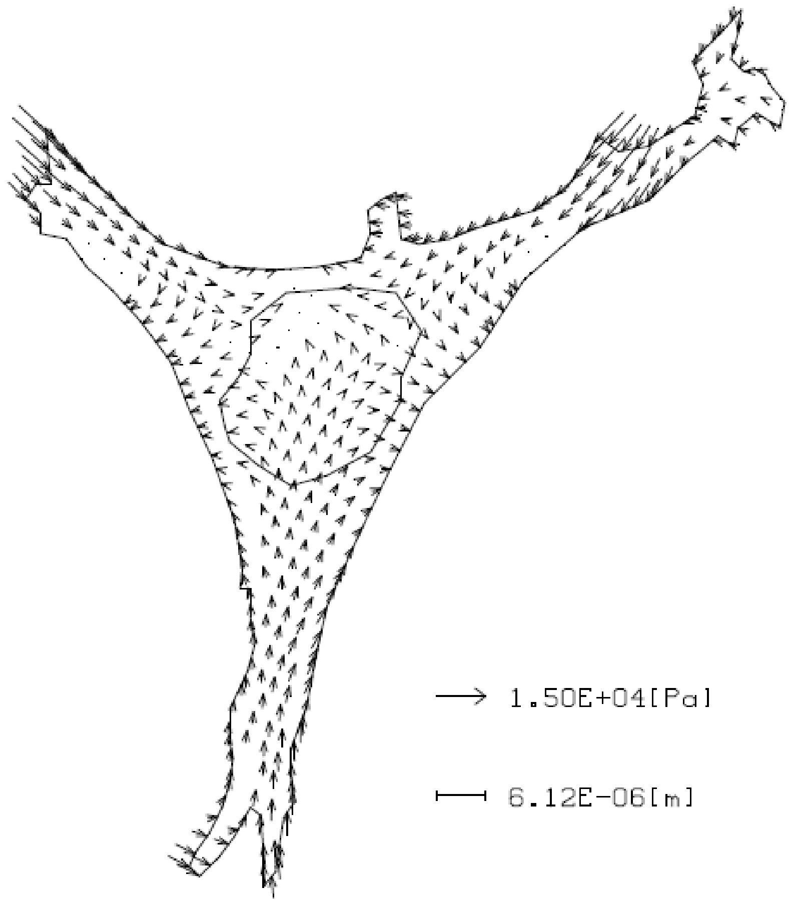

Figure S1. Representative vector plot depicts the magnitude and direction of traction stress exerted by a wild-type MEF cell, The vectors indicate the direction and magnitude of traction stress.

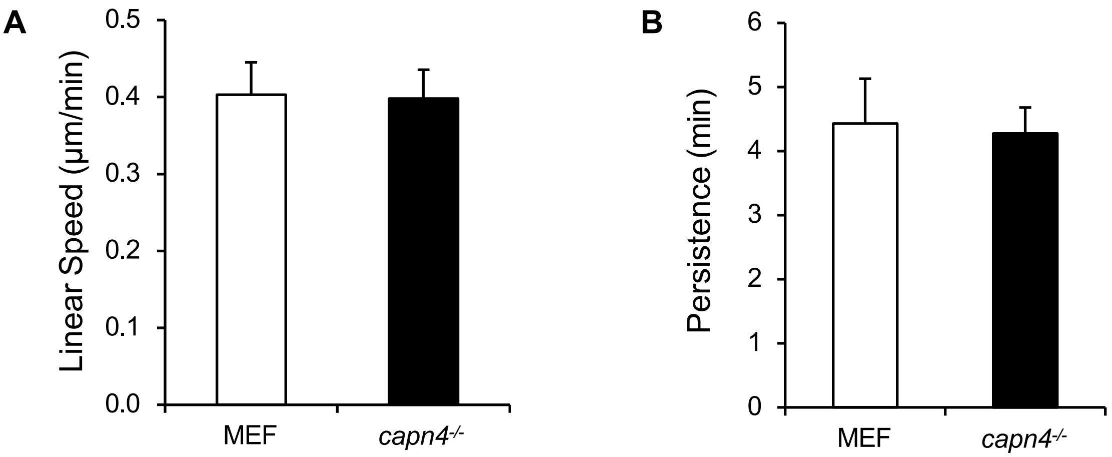

Figure S2. Speed and persistence on Polyacrylamide substrates. *A*) Linear speed of wild-type and *capn4*$^{-/-}$ MEF cells on fibronectin-coated polyacrylamide substrates. (*B*) Persistence of wild- type (n=15) and *capn4*$^{-/-}$ MEF cells (n=15) on fibronectin-coated polyacrylamide substrates. Each cell was imaged 2 hours, each trial 2 cells of each cell type was observed, for a total of 7 trials. Error bars represent mean ± SEM.

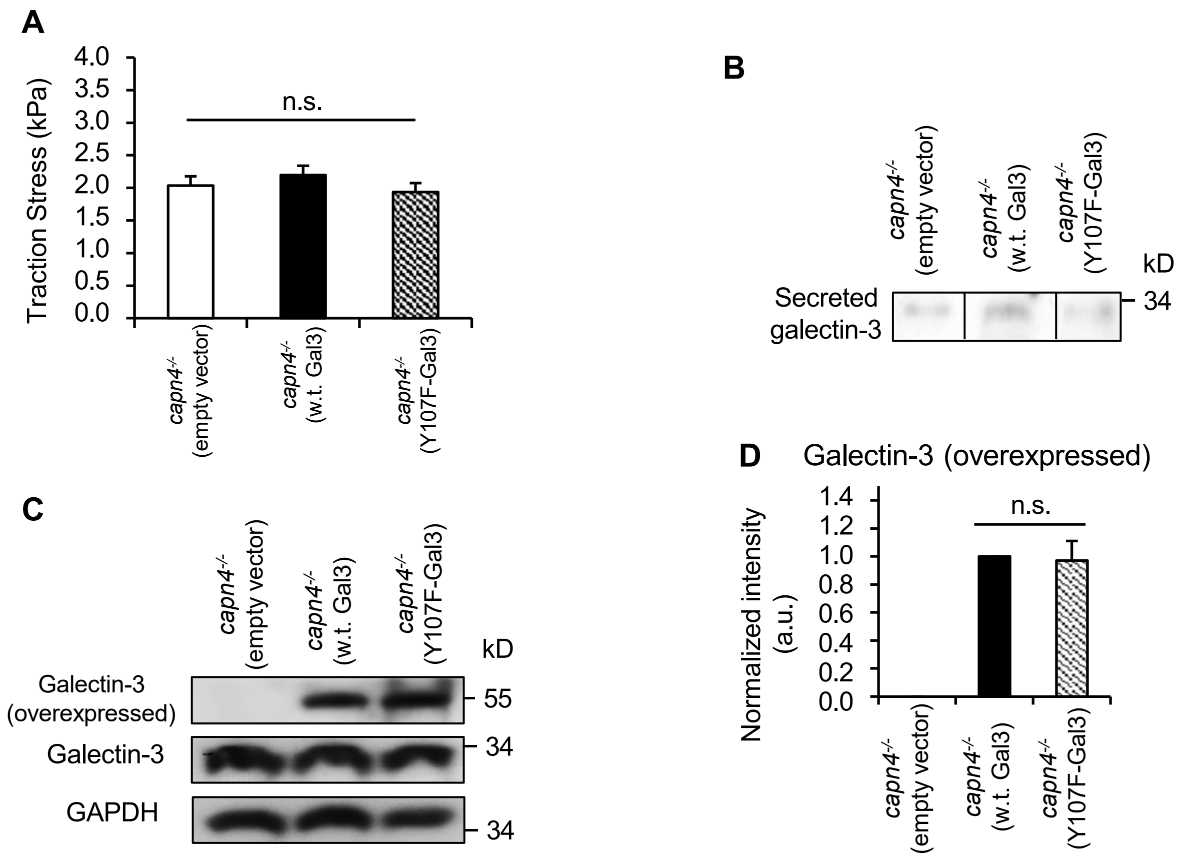

Figure S3. (*A*) Bar graph of average traction stress exerted by *capn4*$^{-/-}$ MEF cells nucleofected with pEGFP-N3 (empty vector; n=19), pECFP-Gal3 (w.t. Gal3; n=15) or pEGFP-Y107F-Gal3 (Y107F-Gal3; n=14). (*B*) Western blot of Gal3 from conditioned media of *capn4*$^{-/-}$ MEF cells with empty vector, w.t. Gal3 or Y107F-Gal3. Quantification of data are not measurable for a graph. (*C*) Western blot of overexpressed and endogenous Gal3 from cell lysates of empty vector, w.t. Gal3 or Y107F-Gal3 in *capn4*$^{-/-}$ MEF cells. GAPDH served as the loading control. (*D*) Quantification of overexpressed Gal3 levels of *capn4*$^{-/-}$ MEF cells with empty vector, w.t. Gal3 or Y107F-Gal3. Normalized intensity expressed in arbitrary units in the bar graphs is an average of three independent experiments. Error bars represent mean ± SEM. n.s., not significant.
